# Heat-Killed *Bifidobacterium longum* KABP-042 and *Lactiplantibacillus Plantarum* KABP-051 Prevent Scopolamine-Induced BDNF Reduction and Memory Impairment in Mice

**DOI:** 10.1007/s12013-026-02057-5

**Published:** 2026-03-14

**Authors:** Mamoru Fukuchi, Naoki Arai, Shinichi Honda, Yuji Tominaga

**Affiliations:** 1 Laboratory of Molecular Neuroscience, Faculty of Pharmacy, Takasaki University of Health and Welfare, 60 Nakaorui-machi, Takasaki, 370- 0033 Gunma Japan; 2 Supplement & Probiotics Research Laboratories, Kaneka Corporation, 1-8, Miyamae-cho, Takasago-cho, Takasago, 676-8688 Hyogo Japan

**Keywords:** brain-derived neurotrophic factor, bioluminescence imaging, heat-killed lactic acid bacteria, scopolamine

## Abstract

Brain-derived neurotrophic factor (BDNF) plays a pivotal role in learning and memory, and reductions in BDNF levels are closely associated with cognitive decline and psychiatric disorders. Dietary factors that maintain or restore brain BDNF expression may therefore contribute to brain health. In this study, we evaluated the effects of heat-killed lactic acid bacteria (LAB) on scopolamine-induced BDNF reduction using *Bdnf-Luciferase* (*Bdnf-Luc*) transgenic mice, which allow noninvasive monitoring of BDNF expression in the living brain by in vivo bioluminescence imaging. Administration of scopolamine induced a significant reduction in brain BDNF expression, which was successfully visualized by bioluminescence imaging. Oral administration of heat-killed *Bifidobacterium longum* KABP-042 markedly prevented this reduction, while heat-killed *Lactiplantibacillus plantarum* KABP-051 showed partial protective effects. Furthermore, both LAB strains significantly ameliorated scopolamine-induced impairment of hippocampus-dependent memory in contextual fear conditioning. These results demonstrate that specific heat-killed LAB exert protective effects against BDNF reduction and cognitive dysfunction in vivo. In addition, our findings highlight the utility of *Bdnf-Luc*-based in vivo bioluminescence imaging as a practical tool for evaluating dietary factors with potential benefits for brain health.

**Supplementary Information:**

The online version contains supplementary material available at https://doi.org/10.1007/s12013-026-02057-5.

## Introduction

Mental disorders, including depression and dementia, represent major global health challenges worldwide [1–6]. These conditions not only impair quality of life but also impose substantial burdens on families, caregivers, and healthcare systems. Although genetic predisposition contributes to disease susceptibility, mental disorders generally arise from complex interactions between genetic and environmental factors [7]. A number of environmental risk factors have been identified [8], and importantly, several of these are potentially modifiable. For example, metabolic dysfunction is associated with depression, insufficient physical activity is linked to Alzheimer’s disease (AD), and childhood adversity increases the risk of schizophrenia spectrum disorders [9]. In addition to minimizing such risk factors, increasing attention has been paid to the functional properties of dietary components as a strategy to support mental health. Nutritional formulations enriched with bioactive compounds–including dietary fibers, polyphenols, omega-3 fatty acids, amino acids, vitamins, and minerals– have been suggested to prevent or delay AD progression, support mental well-being, and help manage psychiatric conditions [10, 11]. Thus, dietary interventions represent a promising and accessible approach to preserving brain health.

In this context, the gut–brain axis has emerged as a key pathway linking dietary factors, intestinal microorganisms, and brain function. Among dietary microorganisms, lactic acid bacteria (LAB) have attracted particular interest due to their long history of safe consumption and well-documented health-promoting properties [12]. The functional characteristics and biotechnological applications of LAB in food systems have been extensively investigated, with safety being one of their most firmly established features [13]. The recognition of many LAB strains as probiotics has further expanded their potential biomedical applications [14, 15]. Importantly, accumulating evidence indicates that specific LAB strains can influence cognitive function and mental health through modulation of the gut–brain axis [16]. Recent studies have also highlighted the beneficial effects of inactivated probiotics on brain function [17]. Such inactivated microorganisms and their bioactive components are classified as postbiotics [18], and both live probiotics and postbiotics have been shown to modulate gut–brain communication [17, 18].

Brain-derived neurotrophic factor (BDNF), a member of the neurotrophin family, plays a central role in synaptic plasticity, learning, and memory [19–21]. High circulating and brain levels of BDNF have been associated with protection against age-related cognitive decline [22, 23]. Conversely, a low serum BDNF level correlates with poor cognitive performance and increased risk of mild cognitive impairment [24]. Moreover, decreased BDNF concentrations in the cerebrospinal fluid predict progression from mild cognitive impairment to AD [25]. Additionally, stress has been shown to suppress BDNF expression [26], and patients with major depressive disorder exhibit low BDNF levels [27]. Postmortem studies suggest that antidepressant treatment can restore BDNF expression [28, 29]. Collectively, these findings highlight BDNF not only as a biomarker for cognitive decline and psychiatric disorders but also as a promising therapeutic target. In the present study, we therefore focused on BDNF as a primary molecular endpoint because it represents an integrative marker linking gut–brain axis [30] as well as synaptic plasticity, learning, and memory. Although other molecular pathways, including CREB signaling, synaptic proteins, neuroinflammatory mediators, and glial activation, are also critically involved in cognitive regulation [17, 31–33], our primary aim was to establish a sensitive and noninvasive in vivo screening platform for dietary factors capable of modulating neurotrophic support in the brain.

Consistent with this concept, accumulating evidence demonstrates that dietary components can modulate BDNF expression [34]. For example, omega-3 fatty acid supplementation normalizes BDNF levels, reduces oxidative stress, and improves learning deficits in rodent models of traumatic brain injury [35]. Similarly, branched-chain amino acids restore BDNF expression and enhance resilience to chronic social defeat stress in mice [36]. These observations underscore the importance of identifying dietary microorganisms and food-derived factors capable of modulating BDNF signaling in vivo.

To facilitate in vivo evaluation of BDNF expression, we previously generated *Bdnf-Luciferase* (*Bdnf-Luc*) mice, a transgenic strain expressing firefly luciferase under the control of the *Bdnf* promoters, enabling monitoring of BDNF expression in living animals [37, 38]. Using the transgenic mice, we successfully visualized activity-regulated BDNF expression in the brains of living mice [37, 39]. This approach allows longitudinal assessment of BDNF dynamics within the same individual and provides a sensitive platform for evaluating environmental and dietary influences on neurotrophic signaling.

In the present study, we applied this established *Bdnf-Luc* mouse system to evaluate the effects of heat-killed LAB on brain BDNF expression. We employed scopolamine, a muscarinic acetylcholine receptor antagonist widely used to model cognitive impairment [40, 41], which has also been reported to reduce BDNF expression in the brain [42, 43]. This is a well-established pharmacological model that reproduces key features of cholinergic dysfunction, memory deficits, and reduced neurotrophic signaling observed in cognitive disorders, and provides a suitable experimental framework for evaluating dietary and postbiotic interventions aimed at preserving brain function [32, 43–45]. Using this model, we demonstrate that oral administration of heat-killed *Bifidobacterium longum* KABP-042 significantly prevented scopolamine-induced BDNF reduction, while heat-killed *Lactiplantibacillus plantarum* KABP-051 exerts partial protective effects. Furthermore, both strains ameliorate scopolamine-induced impairment of hippocampus-dependent memory. These findings identify specific LAB strains as promising postbiotic candidates for supporting brain health and highlight the utility of *Bdnf-Luc*-based bioluminescence imaging as a practical tool for screening dietary factors with neuroprotective potential.

## Materials and Methods

### Reagents

Scopolamine hydrobromide n-hydrate and donepezil hydrochloride were purchased from FUJIFILM Wako Pure Chemical Corporation (Osaka, Japan) and dissolved in saline (Otsuka Pharmaceutical Co., Ltd., Tokyo, Japan) prior to use.

### Animals

**Fig. 1:**
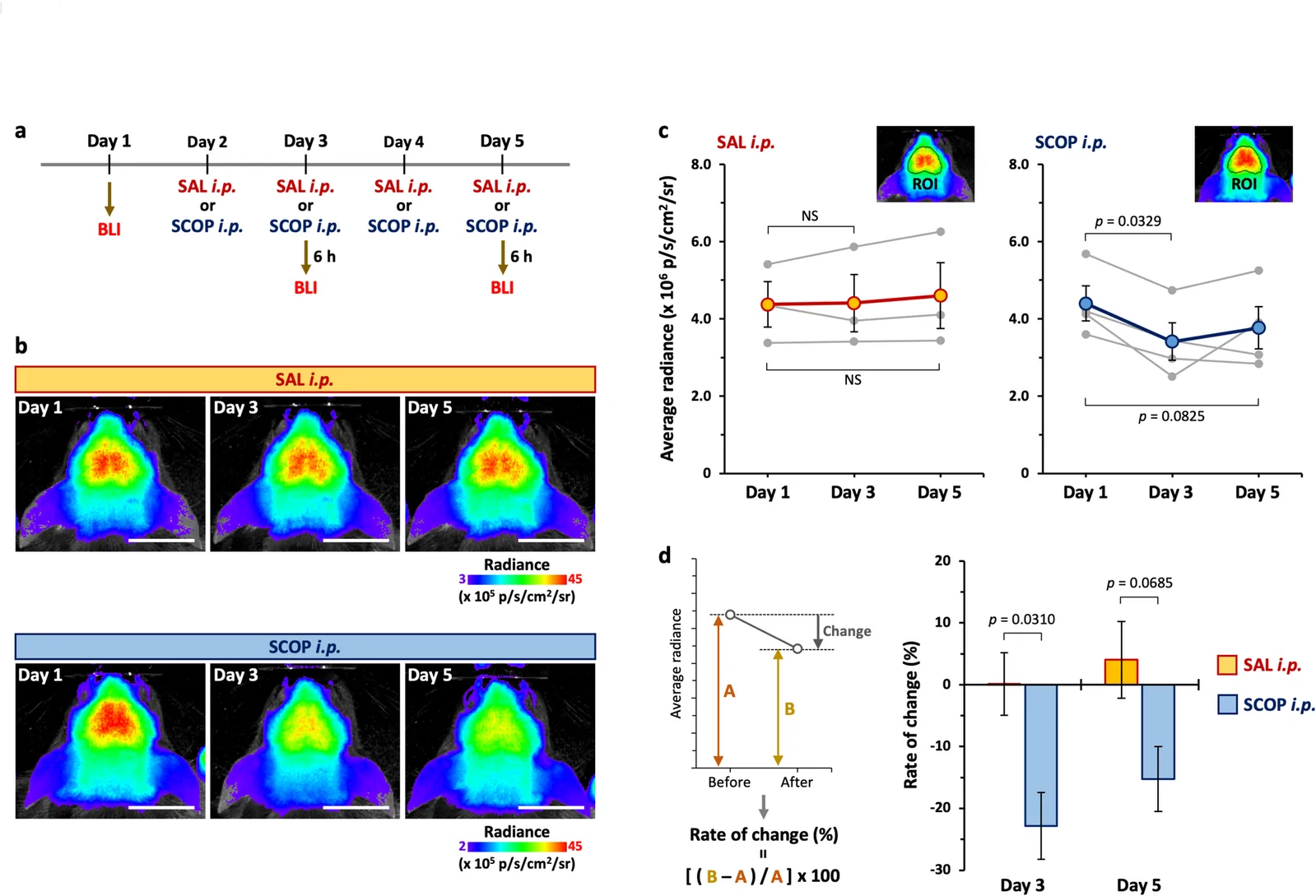
Establishment of bioluminescence imaging (BLI) for detection of scopolamine-induced brain-derived neurotrophic factor (BDNF) reduction in the living mouse brain. (**a**) Experimental schedule. On day 1, BLI was conducted to measure the basal intensity of bioluminescence signals. On days 2, 3, 4, and 5, saline (SAL) or scopolamine (SCOP, 1 mg/kg body weight) was administered intraperitoneally to *Bdnf-Luc* mice. On days 3 and 5, BLI was performed 6 h after the intraperitoneal administration of SAL or SCOP. (**b**) Representative images of the bioluminescence signals. Scale bar = 1 cm. (**c**) Region of interest (ROI) analysis. Data represent the mean ± SEM of three (SAL) or four (SCOP) independent experiments (gray lines indicate individual intensity). Statistical analysis was conducted using repeated measures ANOVA with Dunnett’s multiple comparisons test. (d) (Left) The calculation for the rate of change (%) is shown. (Right) Rates of change (%) after the administration of SAL or SCOP are shown. Data represent the mean ± SEM [n = 3 (SAL) and n = 4 (SCOP)]. Statistical analysis was conducted using two-way ANOVA with Sidak’s multiple comparisons test

All animal procedures were approved by the Animal Experiment Committee of Takasaki University of Health and Welfare (Authorization Nos. 2008 and 2513) and were conducted in accordance with the Guidelines for the Care and Use of Laboratory Animals of Takasaki University of Health and Welfare. Mice were maintained under standard laboratory conditions (12 h–12 h/light–dark cycle at 22 ± 2 °C) with ad libitum access to food and water. The generation of *Bdnf-Luc* mice has been described previously [38]. For the experiments shown in Fig. 1 and Supplementary Fig. S1, male and female *Bdnf-Luc* mice (2–4 months old) were used. The other experiments were conducted using 2 months old male *Bdnf-Luc* mice.

**Fig. 2:**
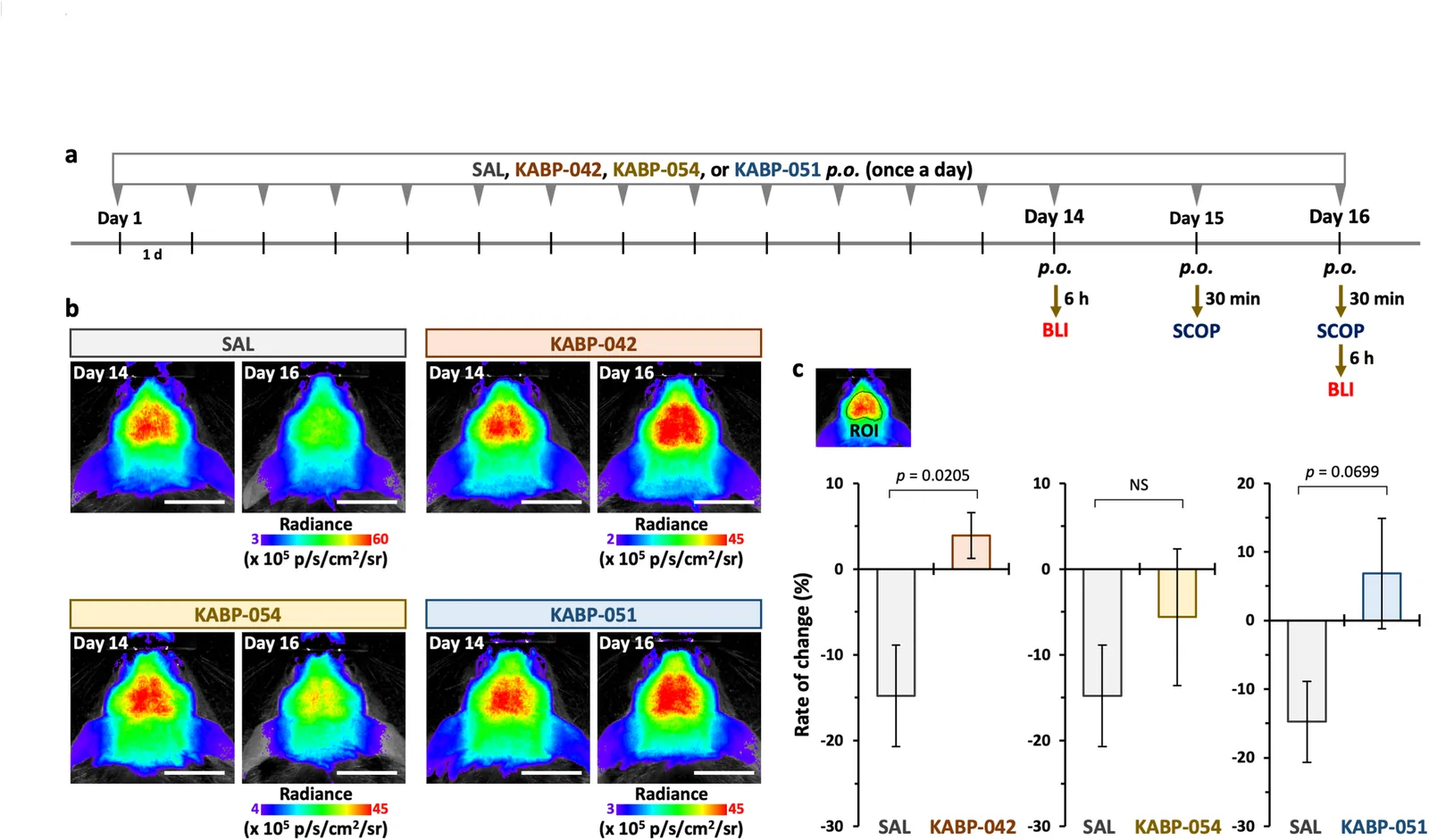
Effects of heat-killed LAB on scopolamine-induced BDNF reduction in the brain.(**a**) Experimental schedule. Saline (SAL) or heat-killed LAB (KABP-042, KABP-054, or KABP-051) were administered orally to *Bdnf-Luc* mice for 14 consecutive days (once daily). On day 14, BLI was conducted to measure the signal intensity before scopolamine (SCOP) injection. On days 15 and 16, SCOP (1 mg/kg body weight) was administered intraperitoneally to *Bdnf-Luc* mice 30 min after oral administration of SAL or LAB. On day 16, BLI was performed 6 h after the intraperitoneal administration of SCOP. (**b**) Representative images of the bioluminescence signals before and after SCOP injection. (**c**) Rate of change (%) after SCOP injection. Data represent the mean ± SEM [n = 3 (KABP-054 and KABP-051) and n = 5 (SAL and KABP-042)]. Statistical analysis was conducted using an unpaired t test

**Fig. 3:**
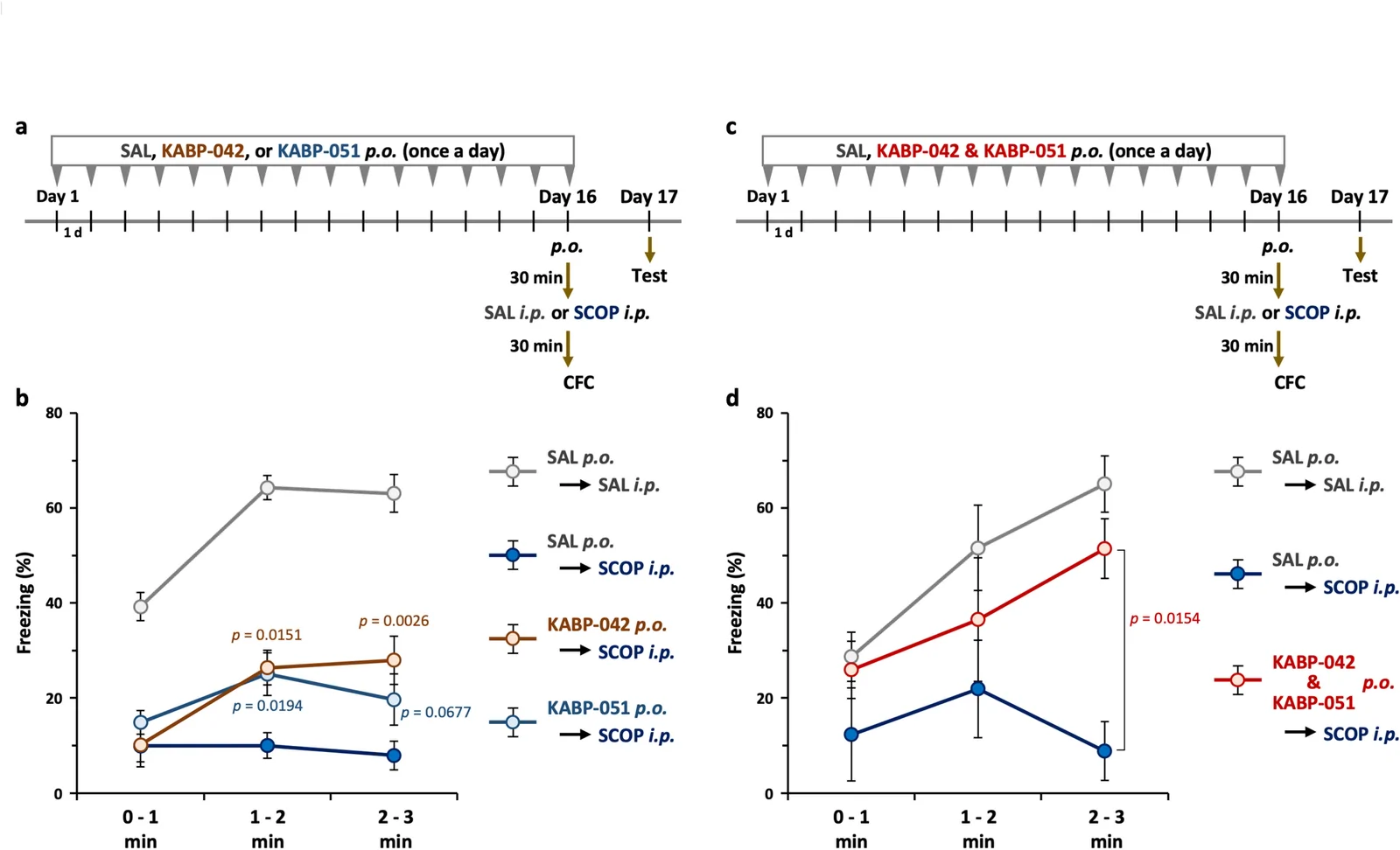
Effects of heat-killed LAB on scopolamine-induced hippocampus-dependent memory impairment in mice. (**a**) Experimental schedule. Saline (SAL) or heat-killed LAB (KABP-042 or KABP-051) was administered orally to C57BL/6 N mice for 16 consecutive days (once daily). On day 16, SAL or scopolamine (SCOP, 2 mg/kg body weight) was administered intraperitoneally to the mice 30 min after the oral administration of SAL or LAB, and contextual fear conditioning was conducted 30 min after the intraperitoneal injection. One day after contextual fear conditioning, the mice were exposed to the same conditioning chamber (test). (**b**) Freezing behavior of the mice in 3-min test sessions. The results show changes in freezing (%) every min during the 3-min test. Data represent the mean ± SEM [n = 8 (SAL and KABP-042) and n = 10 (KABP-051)]. Statistical analysis was conducted using two-way ANOVA with Dunnett’s multiple comparisons test (Comparisons among three groups of mice administered SCOP following treatment with SAL, KABP-042, or KABP-051). (**c**) Experimental schedule. SAL or a mixture of heat-killed KABP-042 and KABP-051 (KABP-042 & KABP-051, 1:1 ratio based on cell count) was administered orally to C57BL/6 N mice for 16 consecutive days (once daily). The dosage was approximately 2 × 109 bacterial cells/day (1 × 109 bacterial cells of each strain). On day 16, SAL or SCOP (2 mg/kg body weight) was administered intraperitoneally to the mice 30 min after oral administration of SAL or LAB, and contextual fear conditioning was conducted 30 min after the intraperitoneal injection. One day after the contextual fear conditioning, the mice were exposed to the same conditioning chamber (test). (**d**) Freezing behavior of the mice in 3-min test sessions. The results show changes in freezing (%) every min during the 3-min test. Data represent the mean ± SEM [n = 3 (SAL) and n = 4 (KABP-042 & KABP-051)]. Statistical analysis was conducted using two-way ANOVA with Sidak’s multiple comparisons test (Comparisons between two groups of mice administered SCOP following treatment with SAL or a mixture of KABP-042 and KABP-051)

Male C57BL/6 N mice (6 weeks old) were purchased from Japan SLC (Shizuoka, Japan) and acclimatized for approximately 2 weeks before being used in experiments. To confirm the robustness of the bioluminescence imaging system, we used both male and female *Bdnf-Luc* mice in our initial imaging experiments (Fig. 1 and Supplementary Fig. S1). In subsequent experiments, we planned to use LAB-treated mice for behavioral analysis using contextual fear conditioning tests (Fig. 3 and Supplementary Figs. S2 and S3) in addition to imaging (Fig. 2). Because sex differences in contextual fear conditioning have been reported, with several studies suggesting lower freezing responses in females compared to males [46], we used only male mice in the present study (except for the experiments shown in Fig. 1 and Supplementary Fig. S1) to reduce behavioral variability and enhance experimental consistency.

### Drug Administration

Scopolamine hydrobromide n-hydrate was dissolved in saline to a concentration of 0.1 mg/mL (Figs. 1 and 2 and Supplementary Figs. S1 and S2a) or 0.2 mg/mL (Fig. 3 and Supplementary Figs. S2b and S3). Donepezil hydrochloride was dissolved in saline to a concentration of 0.5 mg/mL. These solutions were prepared prior to injection. The volume of administration was 0.1 mL/10 g body weight. Scopolamine hydrobromide n-hydrate was administered intraperitoneally at doses of 1 mg/kg (Figs. 1 and 2 and Supplementary Figs. S1 and S2a) or 2 mg/kg body weight (Fig. 3 and Supplementary Figs. S2b and S3). Donepezil hydrochloride was administered intraperitoneally at 5 mg/kg body weight. Drug administration timing relative to bioluminescence imaging and behavioral analysis is described in the corresponding figures and figure legends (Figs. 1a, 2a and 3a, and 3c, and Supplementary Figs. S1a, S1d, S2, and S3a).

### Bioluminescence Imaging

Bioluminescence imaging (BLI) was performed as described previously [37, 39, 47]. The black fur of the mice was shaved 1 day prior to BLI. Under inhalation anesthesia with 2% isoflurane, *Bdnf-Luc* mice were intraperitoneally injected with TokeOni (Kurogane Kasei Co., Ltd., Aichi, Japan) at a dose of 100 mg/kg. BLI was conducted using an IVIS in vivo imaging system (PerkinElmer, Boston, MA, USA) at 4–6 min after injection and repeated every 2 min to obtain peak signal intensity. Regions of interest (ROIs) were defined to encompass the whole brain area visible from the dorsal aspect of the head, as shown in the figures (The area enclosed by a black line in each figure), and signal intensities were quantified using the Living Image software (PerkinElmer) under identical settings for all experimental groups.

### Cultivation of LAB

*Lactiplantibacillus plantarum* KABP-051 and *Streptococcus dentisan*i KABP-054 were cultured in Man-Rogosa-Sharpe broth under aerobic conditions. *Bifidobacterium longum* KABP-042 was cultured in Man-Rogosa-Sharpe broth supplemented with 0.05% (w/v) cysteine-HCl under anaerobiosis. All cell culture was conducted at 37 °C for 24 h. Bacterial cells amplified in culture broth were harvested by centrifugation at 18,800 × g for 10 min at 4 °C, then washed two times with sterilized water. Bacterial cells resuspended with sterilized water at a concentration of approximately 2 × 10^10^ colony-forming units/mL were heat-killed by heating at 90 °C for 30 min in a water bath. The bacterial cell suspension was frozen in a − 80 °C freezer and freeze-dried to prepare heat-killed bacteria. The freeze-dried bacterial cells were sifted through a wire mesh to produce fine powder and stored at − 80 °C.

### Administration of Heat-Killed LAB to Mice

The freeze-dried powder of heat-killed LAB was resuspended in saline prior to use. The suspension was administered orally to mice once daily. The dosage was approximately 1 × 10^9^ bacterial cells/day. The schedule for the administration of heat-killed LAB is shown in the corresponding figures.

### Contextual Fear Conditioning

Hippocampus-dependent memory was assessed using contextual fear conditioning. Mice were placed in a conditioning chamber (width 30 cm × depth 24 cm × height 21 cm) with a grid floor capable of delivering electrical foot shocks. The chamber was illuminated at an intensity of approximately 330–340 lx during the experiment. On the training day, mice were placed into the chamber, and after 148 s received a 2-s foot shock (0.4 mA). They were returned to their home cage 30 s after the foot shock. Twenty-four hours after training, the mice were re-exposed to the chamber for 3 min, and freezing responses were automatically quantified using a video-based system (FreezeFrame-4, Actimetrics, Lafayette, IN, USA).

### Statistical Analysis

All data are presented as mean values ± standard error of the mean (SEM). Statistical analyses were performed using Prism 7 software (GraphPad, Boston, MA, USA) as described in the figure legends. Statistical significance was defined as *p* < 0.05.

## Results

### Establishment of BLI for Visualizing Scopolamine-Induced Reduction of BDNF Expression

We employed *Bdnf-Luc* mice to monitor changes in BDNF expression following scopolamine administration. As shown in Fig. 1a day before the administration of scopolamine, BLI was conducted to measure the bioluminescence signal intensity under basal conditions (day 1). After BLI, scopolamine was administered intraperitoneally to the *Bdnf-Luc* mice once daily at a dose of 1 mg/kg for 4 consecutive days. BLI was performed again 2 or 4 days after the first BLI session [day 3 and day 5, respectively (Fig. 1a)]. Saline was also injected into the control *Bdnf-Luc* mice. As shown in Fig. 1b, the bioluminescence signals were reduced by scopolamine administration, whereas saline injection produced only a small effect on the signals. Region of interest analysis confirmed that scopolamine significantly decreased signal intensities, whereas saline had no effect (Fig. 1c). Calculation of the rate of change further demonstrated a significant signal reduction in scopolamine-treated mice (Fig. 1d). However, it is likely that a single administration of scopolamine produced little effect (Supplementary Fig. S1a–c). Consistent with previous reports that donepezil, an acetylcholinesterase inhibitor, rescues scopolamine-induced reduction of BDNF expression [41], co-administration of donepezil prevented a reduction in bioluminescence signals (Supplementary Fig. S1d–f). On the basis of these results, we adopted a protocol consisting of scopolamine administration once daily for 2 consecutive days at 1 mg/kg to reliably induce measurable BDNF reduction (Fig. 1d).

### Effects of Oral Administration of Heat-Killed LAB on Scopolamine-Induced BDNF Reduction

We next investigated whether heat-killed LAB could prevent scopolamine-induced suppression of BDNF expression (Fig. 2a). Mice received daily oral administration of heat-killed *Bifidobacterium longum* KABP-042, *Streptococcus dentisani* KABP-054, and *Lactiplantibacillus plantarum* KABP-051 for 14 consecutive days. On day 14, BLI was conducted 6 h after LAB administration. Scopolamine was injected into the *Bdnf-Luc* mice 30 min after the oral administration of saline or heat-killed LAB on days 15 and 16, and BLI was performed 6 h after the final injection of scopolamine on day 16 (Fig. 2a). As expected, saline-treated mice exhibited a reduction in bioluminescence intensity after scopolamine injection (Fig. 2b). A similar reduction was observed in mice administered KABP-054 (Fig. 2b). In contrast, oral administration of KABP-042 and KABP-051 suppressed scopolamine-induced signal reduction (Fig. 2b). Quantitative analysis confirmed a significant protective effect of KABP-042 (Fig. 2c). These results suggest that KABP-042, and possibly KABP-051, prevent scopolamine-induced decreases in BDNF expression in vivo.

### Effects of Oral Administration of Heat-Killed LAB on Scopolamine-Induced Memory Impairment

We next examined whether heat-killed LAB could ameliorate scopolamine-induced memory impairment. Because our present data suggested that KABP-042, and possibly KABP-051, prevented scopolamine-induced reduction of BDNF expression in the brain (Fig. 2), we particularly focused on the effects of these LAB. In this study, we conducted contextual fear conditioning, which is used to evaluate hippocampus-dependent memory [48]. We first conducted the behavioral test following the same schedule used for BLI, as shown in Fig. 2 (Supplementary Fig. S2a). However, in this experiment, repeated administration of scopolamine at 1 mg/kg, which was sufficient to induce a measurable reduction in BDNF expression detected by bioluminescence imaging, did not reliably impair contextual fear memory under the present experimental schedule (Supplementary Fig. S2a). In contrast, a single administration of scopolamine at 2 mg/kg robustly induced memory impairment (Supplementary Fig. S2b). Therefore, the higher dose was adopted for behavioral analyses to ensure reproducible detection of hippocampus-dependent memory deficits. As shown in Fig. 3b, we found that both KABP-042 and KABP-051 partially but significantly attenuated scopolamine-induced memory deficits. Donepezil also partially rescued freezing behavior (Supplementary Fig. S3). Additionally, oral administration of a mixture of KABP-042 and KABP-051 (Fig. 3c), at a total dose equivalent to the sum of the individual doses, resulted in greater efficacy against memory impairment than administration of either strain alone. These results suggest that oral administration of KABP-042 and KABP-051 ameliorates scopolamine-induced hippocampus-dependent memory impairment.

## Discussion

Accumulating evidence indicates that reduced BDNF expression is closely associated with psychiatric and neurodegenerative disorders, and that restoration of BDNF signaling may contribute to improved cognitive function. In the present study, we applied an in vivo bioluminescence imaging approach using *Bdnf-Luc* mice to visualize scopolamine-induced reductions in brain BDNF expression and to evaluate heat-killed LAB capable of modulating this response. By applying this system, we identified that heat-killed *Bifidobacterium longum* KABP-042, and possibly *Lactiplantibacillus plantarum* KABP-051, can prevent BDNF reduction and partially improve memory deficits in mice.

While a single injection of scopolamine likely produced minimal changes in bioluminescence signals in *Bdnf-Luc* mice, repeated injection (2 consecutive days) reliably induced measurable reductions. Our previous in vitro analysis indicated that the intensities of the bioluminescence signals were not changed within 12 h after the inhibition of transcription with actinomycin D, despite the decreases in luciferase and endogenous *Bdnf* mRNA under this condition [49]. One plausible reason could be the stable expression of firefly luciferase protein, that is, degradation of the luciferase protein could be slower than that of the mRNA, probably resulting in little change in the bioluminescence signal after a single injection of scopolamine. Because endogenous BDNF reduction has been reported to peak at 6 h and seems to be restored by 24 h after scopolamine injection [43], repeated scopolamine administration likely provides the temporal window necessary for reliable detection of the signal reduction. Additionally, although different scopolamine dosing regimens were used for bioluminescence imaging (Fig. 2a) and behavioral analyses (Fig. 3a, c), this may reflect differences in the sensitivity required to detect molecular versus behavioral outcomes.

BDNF is critically involved in distinct phases of memory processing, including acquisition, consolidation, and extinction. BDNF expression is dynamically regulated, with increases during these memory processes [19, 50]. Thus, alterations in hippocampal BDNF levels are closely linked to the efficiency and stability of memory formation and updating. Accumulating evidence indicates that gut microbiota and microbiota-derived factors influence learning and memory, potentially by modulating neurotrophic signaling pathways [51]. In this context, our present study provides a unique experimental framework by enabling noninvasive, longitudinal visualization of BDNF expression in the living brain. Unlike conventional endpoint analyses, the *Bdnf-Luc* system allows assessment of BDNF dynamics within the same individual across different stages of cognitive challenge, thereby capturing experience-dependent changes in neurotrophic signaling. Importantly, the prevention of scopolamine-induced BDNF reduction observed in the present study was accompanied by partial preservation of hippocampus-dependent memory performance, supporting a functional association between in vivo BDNF dynamics and behavioral outcomes. Although further investigations are necessary to demonstrate a causal relationship between BDNF dynamics and behavioral outcomes, these findings suggest that restoration of BDNF signaling may contribute to the observed behavioral protection and highlight the utility of longitudinal BDNF monitoring for linking neurotrophic dynamics with memory-related processes in living animals.

Although the precise mechanisms by which KABP-042 and KABP-051 restore BDNF expression remain unclear, several possibilities are supported by prior studies. The vagus nerve is known to regulate hippocampal BDNF expression, with subdiaphragmatic vagotomy reducing BDNF expression and chronic vagus nerve stimulation increasing it [52, 53]. Glucagon-like peptide-1 (GLP-1) is a candidate for transmission of information between the viscera and brain [54]. GLP-1 is a gut hormone that is secreted from gut epithelial cells in response to the diet. The GLP-1 receptor is highly expressed in the nodose ganglion of the vagus nerve, which relays signals to the nucleus of the solitary tract in the brainstem and further to the higher brain regions. Therefore, secreted GLP-1 from the gut epithelial cells would affect brain functions via activation of the vagus nerve. Additionally, because GLP-1 receptors are expressed in brain regions, including the hippocampus, the peptide could directly modulate brain functions. We found that KABP-042 promoted GLP-1 secretion from murine enteroendocrine STC-1 cells (Supplementary Fig. S4). Although less effective than KABP-042, KABP-051 also showed a tendency to increase slightly GLP-1 secretion (Supplementary Fig. S4). The weaker protective effect observed with KABP-051 compared to KABP-042 might be attributable, at least in part, to differences in their ability to stimulate GLP-1 secretion. Additionally, this GLP-1 secretion was observed with heat-killed cells, indicating that viability and gut colonization were not required. It has been suggested that polysaccharides and other components of heat-killed bacteria might induce hormone secretion from enteroendocrine cells in the intestinal epithelium [55, 56]. Serotonin is another candidate mediator, as gut-derived serotonin also activates vagus afferents and modulates brain function mediated via the nucleus of the solitary tract in the brainstem [57]. Previous studies have shown that heat-killed *Lactobacillus brevis* SBC 8803 enhances serotonin release from intestinal cells [58], increases hippocampal neurogenesis, and improves hippocampus-dependent memory in mice [59]. Although these observations and reports suggest a possible involvement of gut-derived factors, such as GLP-1 and serotonin, this pathway was not directly examined in the present study and should be regarded as a hypothetical mechanism requiring further experimental validation.

We also found that both KABP-042 and KABP-051 partially ameliorated scopolamine-induced impairment of hippocampus-dependent memory. In the present study, not only KABP-042 and KABP-051 but also donepezil exhibited only partial protective effects against scopolamine-induced memory impairment. This limited efficacy may be attributable to the relatively high dose of scopolamine used in our experiments. Previous studies have demonstrated that memory deficits induced by scopolamine at a lower dose (1 mg/kg, half of the dose we employed) can be completely reversed by donepezil [60]. Nevertheless, our findings suggest that the LAB examined here exert effects comparable to those of donepezil in mitigating scopolamine-induced memory impairment. Additionally, oral administration of the KABP-042 and KABP-051 mixture exhibited more protective effects against scopolamine-induced memory impairment. The enhanced efficacy observed with the combined administration of KABP-042 and KABP-051 may reflect additive or complementary mechanisms. One possibility is that co-administration further augments gut hormone secretion, including GLP-1, beyond the level achieved by either strain alone. Alternatively, the two strains may engage partially distinct signaling pathways that converge on memory processes. On the other hand, our current study did not assess neuroinflammatory markers or glial activation in the brain. Given that postbiotics can alleviate neuroinflammatory pathways, glial activation, and cognitive decline in mouse models for dementia [17, 32], it is suggested that modulation of inflammation and glial activation contributes to the observed effects of heat-killed LAB in this study. In any case, further investigation using other model animals would be necessary to ensure the protective effects of these LAB on memory impairment.

Many interventions that restore BDNF expression, including *Angelica keiskei* [45], SKF83959 [60], *Lactobacillus johnsonii* CJLJ103 [44], isoorientin [61], *Scrophularia buergeriana* extract [62], and rutin hydrate [63], have similarly improved memory performance in scopolamine models. Therefore, the restoration of BDNF levels by KABP-042 and KABP-051 may contribute to ameliorating scopolamine-induced memory deficits in mice. Further investigations will be necessary to demonstrate the mechanisms underlying the effects of these LAB on the suppression of scopolamine-induced hippocampus-dependent memory deficits as well as the restoration of BDNF levels. Postbiotic preparations have advantages in safety, stability and versatile applications compared to probiotics, which facilitate their use in various food products, including dietary supplements, beverages and nutraceuticals [64]. These findings in model animals highlight the potential for heat-killed KABP-042 and KABP-051 to provide beneficial interventions in humans.

## Conclusion

In this study, we applied an established in vivo bioluminescence imaging system using *Bdnf-Luc* mice as a platform to evaluate heat-killed LAB that modulate brain BDNF expression. We identified heat-killed *Bifidobacterium longum* KABP-042 as an effective postbiotic that prevents BDNF reduction, while heat-killed *Lactiplantibacillus plantarum* KABP-051 exhibited partial protective activity. Both strains also ameliorated hippocampus-dependent memory impairment in a scopolamine model. These findings demonstrate that specific LAB strains, even in their heat-killed form, can exert biologically relevant effects on brain function. This approach enabled noninvasive visualization of scopolamine-induced BDNF reduction in living mice, and our results highlight the utility of *Bdnf-Luc*-based bioluminescence imaging as a screening tool for identifying dietary factors that support brain health. This platform may facilitate the discovery of functional food ingredients aimed at preserving BDNF expression and mitigating cognitive decline associated with stress and aging.

## Supplementary Information

Below is the link to the electronic supplementary material.

Supplementary Material 1

## Data Availability

The original contributions presented in the study are included in the article/Supplementary Material, further inquiries can be directed to the corresponding authors.
