# Supplementary material for: Heat-Killed Bifidobacterium longum KABP-042 and Lactiplantibacillus Plantarum KABP-051 Prevent Scopolamine-Induced BDNF Reduction and Memory Impairment in Mice
Source: Cell Biochem Biophys. 2026 Mar 14;84(3):3425–35. doi: 10.1007/s12013-026-02057-5 (PMC13585778; doi:10.1007/s12013-026-02057-5)
Supplement: Supplementary file 1 [file 12013_2026_2057_MOESM1_ESM.pdf]

Supplementary Information

Heat-Killed *Bifidobacterium longum* KABP-042 and *Lactiplantibacillus plantarum* KABP-051 Prevent Scopolamine-Induced BDNF Reduction and Memory Impairment in Mice

Mamoru Fukuchi [1], Naoki Arai [2], Shinichi Honda [2], and Yuji Tominaga [2]

[1] Laboratory of Molecular Neuroscience, Faculty of Pharmacy, Takasaki University of Health and Welfare, 60 Nakaorui-machi, Takasaki, Gunma, 370-0033, Japan

[2] Supplement & Probiotics Research Laboratories, Kaneka Corporation, 1-8, Miyamae-cho, Takasago-cho, Takasago, Hyogo, 676-8688, Japan

To whom correspondence should be addressed: Mamoru Fukuchi, Laboratory of Molecular Neuroscience, Faculty of Pharmacy, Takasaki University of Health and Welfare, 60 Nakaorui-machi, Takasaki, Gunma 370-0033, Japan.

E-mail address: fukuchi@takasaki-u.ac.jp

telephone: +81-27-352-1180

fax: +81-27-352-1118

ORCID: 0000-0002-6807-8707 (Mamoru Fukuchi)

Materials and Methods

Cell Culture

Murine enteroendocrine STC-1 cells were cultured in Dulbecco's Modified Eagle Medium (DMEM) (containing 10% fetal bovine serum and 1% Antibiotic-Antimycotic) at 37°C in a 5% CO_2 atmosphere. Cells grown to approximately 70% confluency were detached from the culture dish by trypsin treatment, and a cell suspension adjusted to 4×10^5 cells/mL was seeded at 500 µL per well in a 24-well cell culture plate (2×10^5 cells/well) and cultured for 48 h, which was then used for an *in vitro* assay. After removing the medium from each well, the cells were washed twice with PBS, and 500 µL of reaction solution (DMEM (-) + 1 mM glucose) containing appropriate samples (4 mM glutamate as a positive control, or LAB suspension) was added, and the plate was incubated at 37°C in a 5% CO_2 atmosphere for 6 h. After treatment, the supernatant was collected and immediately frozen at –80°C after spinning down.

Quantification of GLP-1

The quantification of GLP-1 in the reaction solution (supernatant) of cultured cells was performed using a commercial enzyme-linked immunosorbent assay (ELISA) kit [GLP-1 ELISA Kit Wako, high sensitivity (FUJIFILM Wako Pure Chemical Corporation, 299-75501) or GLP-1, Active form (high sensitivity) assay kit (IBL Immuno-Biological Laboratories Co., Ltd., 27700)]. The reaction supernatant was appropriately diluted 20–100 times using the dilution solution included with the kit for measurement.

Supplementary Figure S1

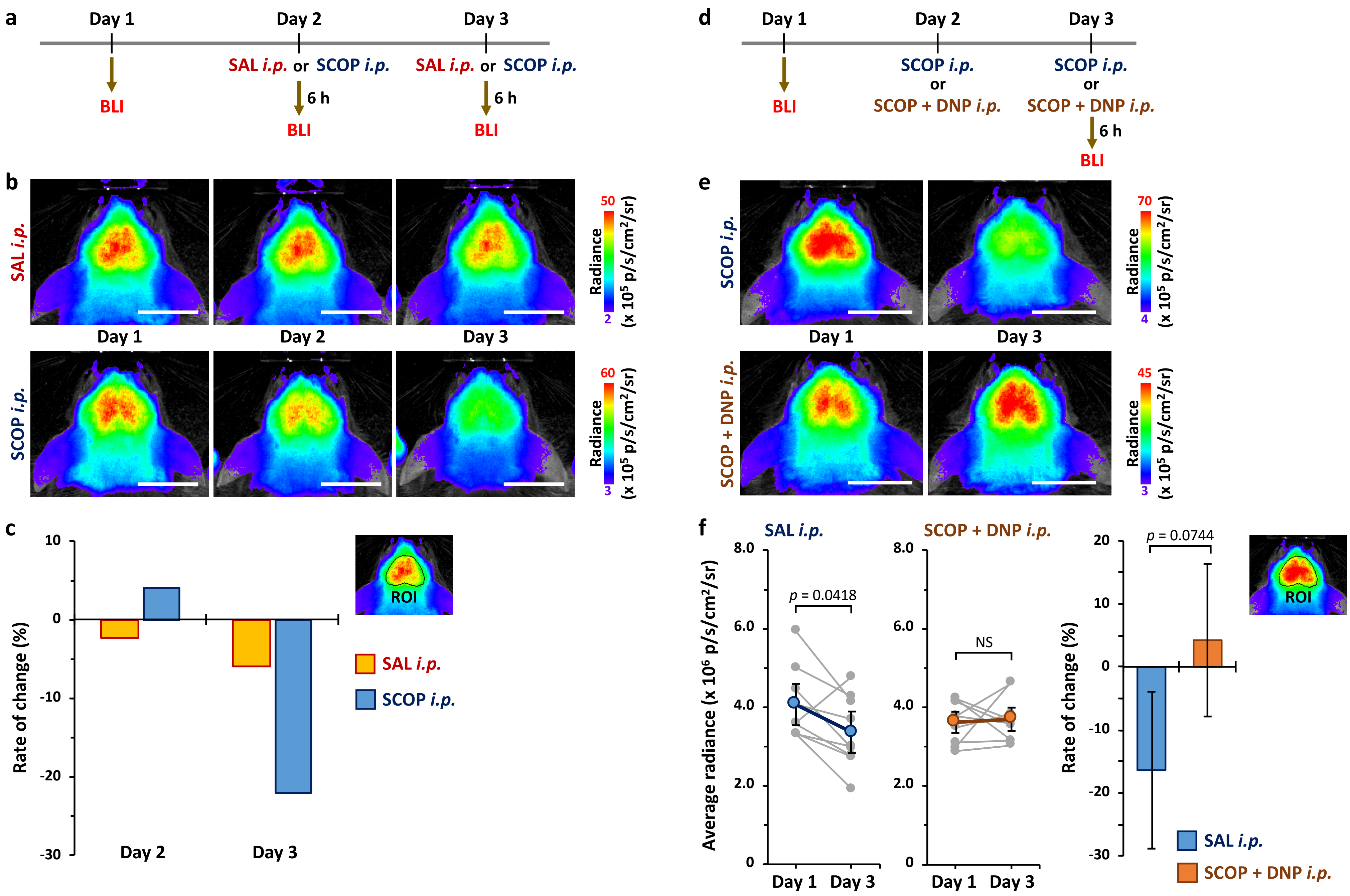

Supplementary Figure S1. Establishment of BLI for detection of scopolamine-induced BDNF reduction in the living mouse brain.

(a) Experimental schedule. On day 1, BLI was conducted to measure the basal intensities of bioluminescence signals. On days 2 and 3, saline (SAL) or scopolamine (SCOP, 1 mg/kg body weight) was administered intraperitoneally to *Bdnf-Luc* mice, and BLI was performed 6 h after the intraperitoneal administration of SAL or SCOP on each day. (b) Representative images of the bioluminescence signals. Scale bar = 1 cm. (c) ROI analysis. Data represent the mean of two independent experiments. (d) Experimental schedule. On day 1, BLI was conducted to measure the basal intensities of bioluminescence signals. On days 2 and 3, SCOP (1 mg/kg body weight) was administered intraperitoneally alone or with donepezil (DNP, 5 mg/kg body weight) to *Bdnf-Luc* mice, and BLI was performed 6 h after the intraperitoneal injection on day 3. (e) Representative images of the bioluminescence signals. Scale bar = 1 cm. (f) (Left) ROI analysis. Data represent the mean ± SEM of nine independent experiments (gray lines indicate individual intensity). The statistical analysis was conducted using a paired *t* test. (Right) Rate of change (%) after the administration of SCOP alone or with DNP. The calculation of the rate of change (%) is shown in Figure 1d. Data represent the mean ± SEM (n = 9). The statistical analysis was conducted using an unpaired *t* test.

Supplementary Figure S2

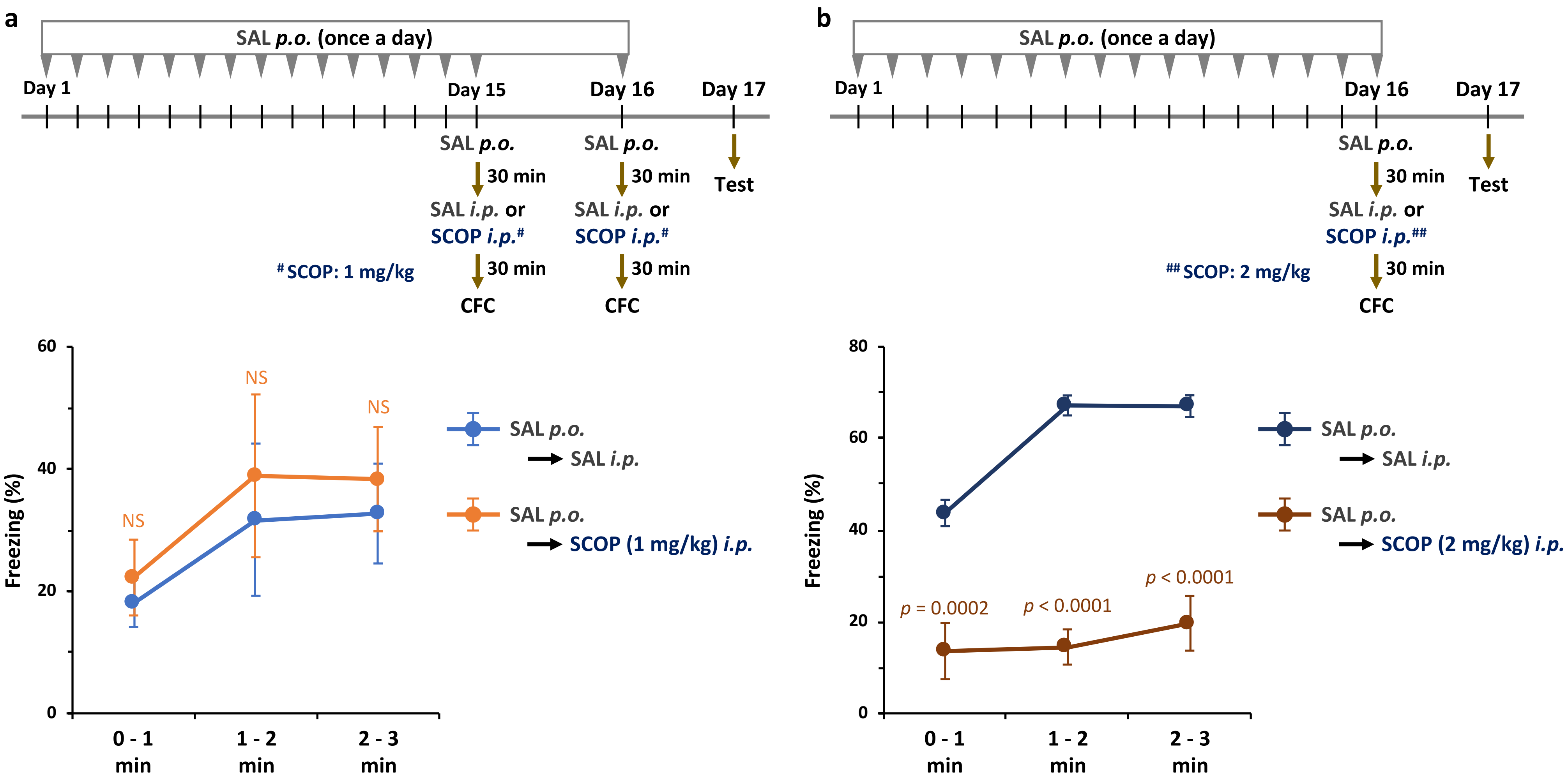

Supplementary Figure S2. Effect of SCOP on freezing behavior after contextual fear conditioning.

(a) SAL was administered orally to C57BL/6N mice for 14 consecutive days. On days 15 and 16, SAL or SCOP (1 mg/kg body weight) was injected intraperitoneally into the mice 30 min after oral administration of saline. On day 16, 30 min after the intraperitoneal injection of SAL or SCOP, contextual fear conditioning was conducted. Freezing behavior was evaluated 1 day after the conditioning. Data represent the mean ± SEM (n = 4). The statistical analysis was conducted using two-way ANOVA with Sidak's multiple comparisons test. NS; not significant. (b) SAL was administered orally to C57BL/6N mice for 15 consecutive days. On day 16, SAL or SCOP (2 mg/kg body weight) was injected intraperitoneally into the mice 30 min after oral administration of SAL, and contextual fear conditioning was conducted 30 min after the injection of SAL or SCOP. Freezing behavior was evaluated 1 day after the conditioning. Data represent the mean ± SEM [n = 5 (SAL) and n = 6 (SCOP)]. The statistical analysis was conducted using two-way ANOVA with Sidak's multiple comparisons test.

Supplementary Figure S3

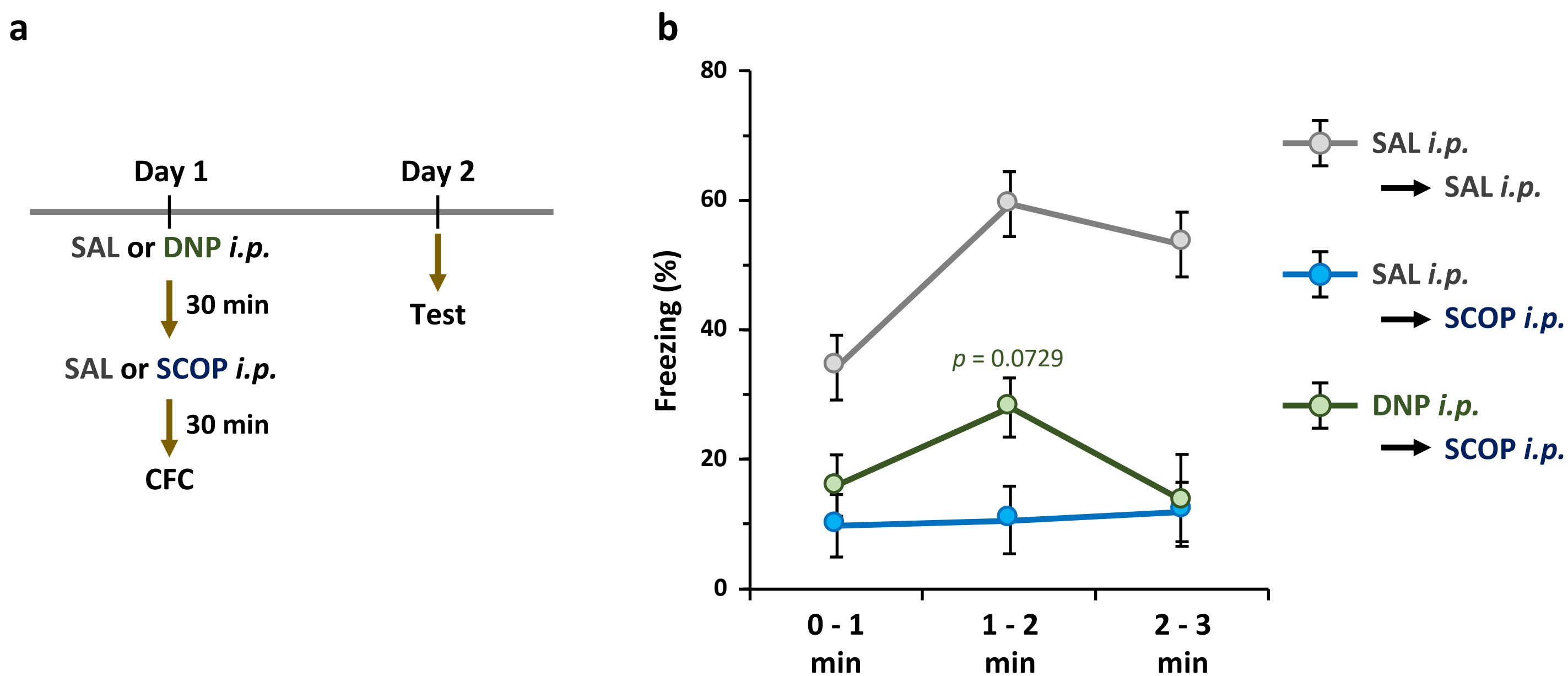

Supplementary Figure S3. Effect of DNP on SCOP-induced impairment of hippocampus-dependent memory.

(a) Experimental schedule. SAL or DNP (5 mg/kg body weight) was injected intraperitoneally into mice. Thirty minutes after the SAL or DNP injection, SAL or SCOP (2 mg/kg body weight) was administered intraperitoneally to the mice, and contextual fear conditioning was conducted 30 min after the final administration. Freezing behavior was evaluated a day after the conditioning. (b) Freezing behavior of the mice in 3-min test sessions. The results show changes in freezing (%) every min during the 3-min test. Data represent the mean ± SEM ($n = 7$). The statistical analysis was conducted using two-way ANOVA with Sidak's multiple comparisons test (Comparisons between two groups of mice administered SCOP following treatment with SAL or DNP).

Supplementary Figure S4

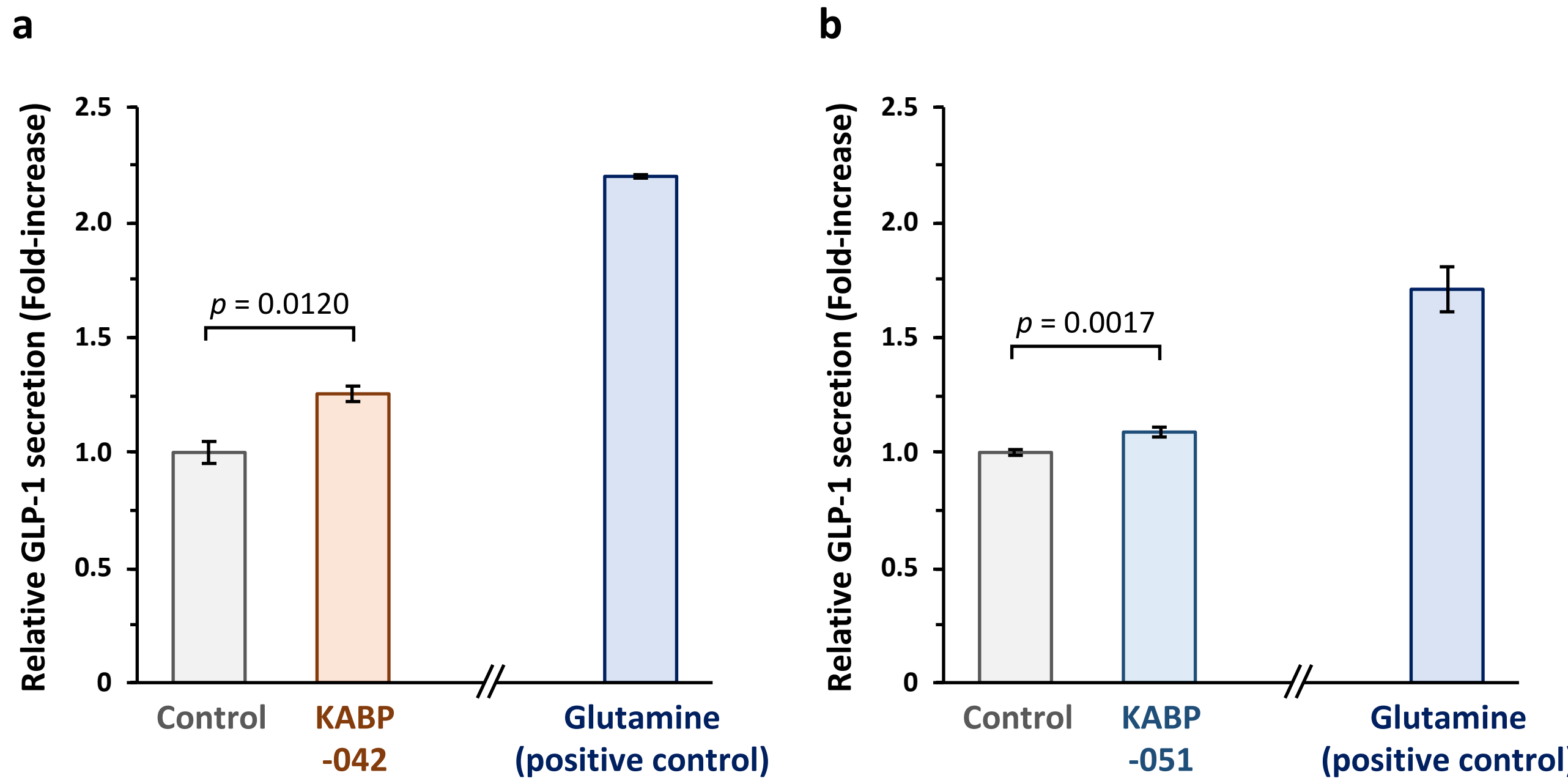

Supplementary Figure S4. Effect of heat-killed LAB on GLP-1 secretion in STC-1 cells.

(a) Relative GLP-1 secretion in STC-1 cells treated with heat-killed cells of KABP-042 at concentration of 30 μg/mL. The amount of GLP-1 after 6 h of reaction was quantified by ELISA. Glutamine (4 mM) was used as a positive control. (b) Relative GLP-1 secretion in STC-1 cells treated with heat-killed cells of KABP-051 at concentration of 3 μg/mL). Data represent the mean ± SEM [n = 3 (a), n = 12 (b)]. The statistical analysis was conducted using an unpaired *t* test.
